# Surface analysis of an eagle talon from Krapina

**DOI:** 10.1038/s41598-020-62938-4

**Published:** 2020-04-14

**Authors:** Davorka Radovčić, Giovanni Birarda, Ankica Oros Sršen, Lisa Vaccari, Jakov Radovčić, David W. Frayer

**Affiliations:** 1 Department of Geology and Paleontology, Croatian Natural History Museum, Zagreb, Croatia; 2 Synchrotron Infrared Source for Spectroscopy and Imaging – SISSI, Elettra - Sincrotrone, Trieste, Italy; 3 Institute for Quaternary Paleontology and Geology, Croatian Academy of Sciences and Arts, Zagreb, Croatia; 4 Department of Anthropology, University of Kansas, Lawrence, KS USA

**Keywords:** Archaeology, Evolution

## Abstract

The Krapina white-tailed eagle talons represent a kind of jewelry worn by Krapina Neandertals some 130,000 years ago. New inspection of one Krapina talon (386.1) revealed a fiber, sealed by a thin silicate coating, adhering to the surface within a wide cut mark, as well as concentrated traces of occasional spots of red and yellow pigment and some black stains. We analyzed the fiber and small portions of pigmented areas by non-invasive, infrared synchrotron beam. Different areas were targeted, revealing the protein nature of the fiber, identified as of animal origin. Targeted areas revealed intra- and inter-strand aggregation indicating the fiber to be collagen losing its original triple α-helix conformation, further confirming the diagenetic decay of the original collagen structure and the antiquity of the fiber. It is possible that the fiber is a remnant of the leather or sinew string binding the talons together. Spectroscopic analysis of the pigments in two isolated areas confirmed two types of ochre and that the dark spots are charcoal remnants. Applying novel non-invasive technologies provides new possibilities to further test the hypothesis of using prehistoric objects for symbolic purposes.

## Introduction

Eight white-tailed eagle (*Haliaëtus albicilla*) talons and one foot phalanx were found at the Krapina Neandertal site, excavated between 1899–1905^1–3^. The deposits are dated to 130,000 BP by ESR and uranium series^4^. Only Neandertals were using the site, evidenced by Mousterian stone tools found in all the Krapina layers, as well as Neandertal remains found in all except the lowest layer. Yet, even here Mousterian stone tools were found in addition to faunal remains with anthropogenic cut marks^5–7^. Deriving from the uppermost level, the talons are thought to be one of the earliest examples of Neandertal ornaments^8^. The fact that eight talons were found in the same context together with an additional phalanx, and the fact that all of them contain evidence of anthropogenic modifications such as cut marks, nicks from the medial and lateral aspects of the plantar surface, and several heavily polished areas suggest that these were an assemblage worn as jewelry. Based on duplication of right talon 2 the talons belong to at least three different birds, indicating that acquiring them was intentional and not a fortuitous capture of a single eagle^8^. This raptor species is a top diurnal avian predator with two-meter wingspan, not commonly encountered in the environment^9^ and not an easy prey to acquire^10,11^.

So far, raptor talons and phalanges have been found at various European Neandertal sites, together with the remains of Mousterian and Châtelperronian techno-cultures^12–17^. All these sites provide evidence of never more than a single talon in the same archaeological level. Only Krapina has multiple talons found in one level with multiple signs of manipulation strongly suggesting they were combined into a personal ornament. They are indeed unique in the European fossil record and, even in the Upper Paleolithic or Mesolithic periods, eight talons from a diurnal raptor in a single level have not been found. Personal ornaments are usually associated with anatomically modern humans^18,19^ and were traditionally recognized as any object prepared for suspension^20,21^. The Krapina eagle talons do not possess drilling that would suggest intentional preparation for suspension in a traditional way for single talons found in much later contexts^22^. However, talons are easy to tie, secure and suspend by binding them around the proximal articulation^23^. Smoothed cut marks, polished areas on lateral plantar edges of all the talons, and nicks on the medial and lateral edges of some talons are all suggestive they were tied around their proximal margins.

**Figure 1:**
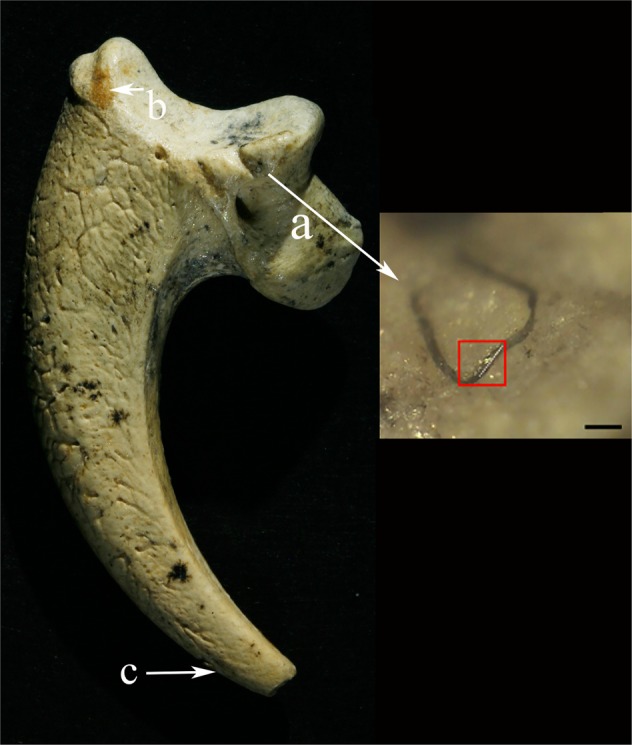
Eagle talon KR386.1. (**a**) Shows the location of the fiber and the enlargement is a closeup of the region. The white dashed line in the area defined by red rectangle represents the portion of the fiber measured by FTIR and SR FTIR. Scalebar is 100 µm. (**b**) The arrow points to the area with the red and yellow ochre mix. (**c**) The arrow indicates the approximate area of black staining.

Recent closer inspection of one talon (386.1) revealed that it possesses evidence of a fiber positioned within a cut mark under a translucent silicate coating (Fig. 1), a result of natural processes and weathering of coarse grained silicoclastics from the cave’s Lower Miocene matrix rock. Small dots of pigments also occur on its surface. These pigments resemble manganese and iron oxides. It is also known that Neandertals used these black and other pigments intentionally, either as staining agents or for large blocks of magnesium oxide as fire starters^24–26^.

To gain better insight into the nature of the fiber and the pigments, it was necessary to use more powerful analytical tools beyond a light microscope. To determine the origin and nature of pigments and the fiber, we searched for non-destructive analyses. It was important to be certain that the surface modifications and the chemical composition of the talon surfaces would not be altered with any analysis. We selected the single talon (386.1) containing both the fiber and pigments and investigated a small portion of this talon focusing on the fiber and pigmented areas using different non-invasive methods. For the fiber analysis, we first tried to determine the nature of the fiber using the scanning elecronic microscope, however this was not conclusive. For the pigments, we first used RAMAN spectroscopy. However, this was unsuccessful due to the high fluorescence signal obscuring the analysis. Therefore, we turned to synchrotron infrared spectrometry.

## Results

**Figure 2:**
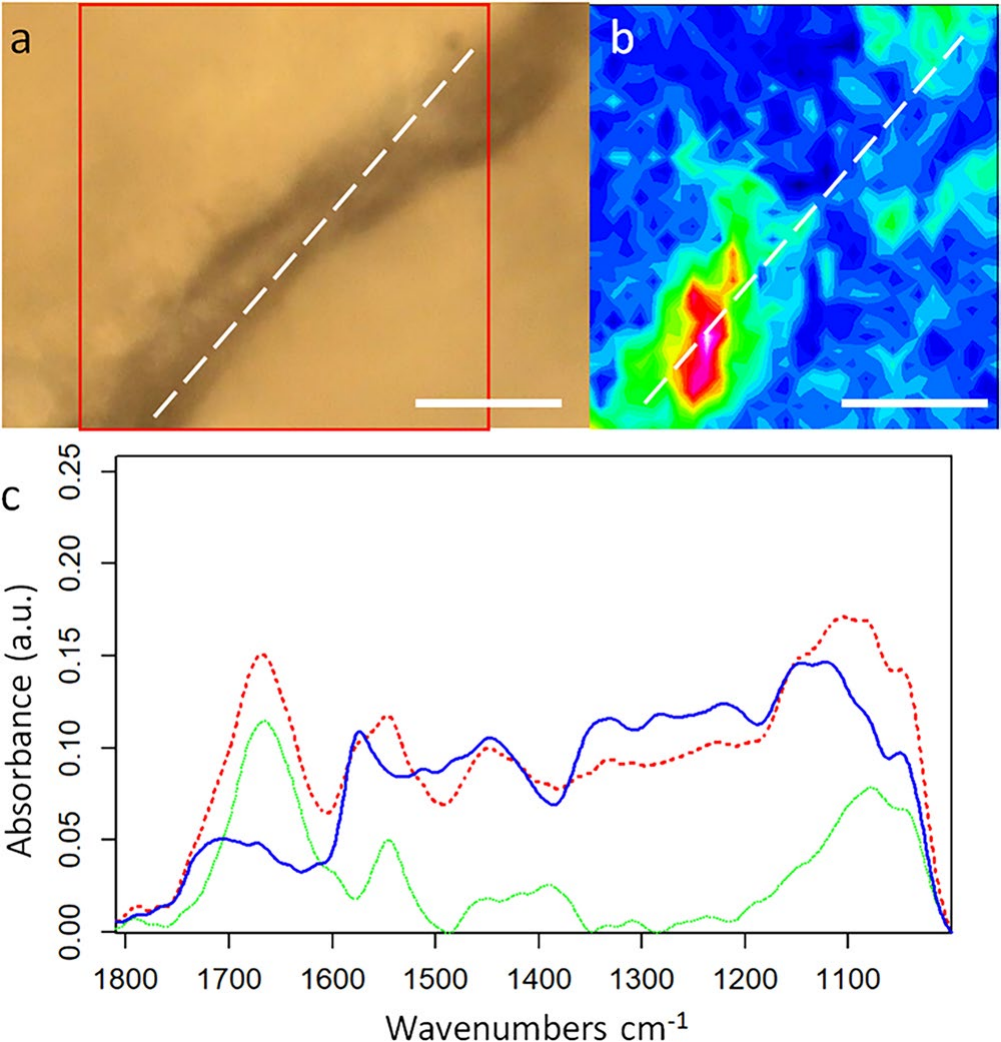
Optical image for fiber. (**a**) Optical image obtained with a cassegrain 15x objective of the inspected fiber on the talon. (**b**) Infrared false color image obtained by the integration of the amide I and amide II bands of the proteins; the scale goes from blue (minimum) to purple-white (maximum). (**c**) Average IR spectrum extracted from the IR map: In red, the average spectrum of the fiber extracted from the IR image, marked as a white dashed line in the panel. In blue, an average spectrum of the area surrounding the fiber. In green, the difference spectrum of the previous two spectra, presenting the main features of the fiber alone, the blue spectrum has been subtracted from the red one with a scaling factor in order to match the signals in the 1300-1150 cm^−1^ spectral region. Scalebars are 50 µm.

The fiber and the pigmented areas are covered by a translucent silicate layer, which preserved the original integrity of the micro-artifacts. This was apparently part of the natural preservation process, which sealed these areas from subsequent contamination. We are uncertain when the talon was sealed by the silicate coating, but it was not recent based on our analysis described below. Fortunately, unlike many of the other Krapina bones, this talon was not covered with shellac, making analysis uncomplicated by post-excavation preservatives. The chemical nature of the fiber was analyzed and visualized by the integration of specific signals of the acquired infrared data. Analysis of the spectral feature of the sample made it possible to exclude a vegetal origin since the spectra were strongly characterized by the presence of amide I and amide II bands. Amide bands are a group of bands characteristic of proteins that are generated by the vibrations of the peptide bond^27^. The peptide group gives up to nine characteristic bands (amide A, B, I, II, III, IV, V, VI, VII). The amide A band (about 3500 cm −1) and amide B (about 3100 cm −1) originate from a Fermi resonance between the first overtone of amide II and the N-H stretching vibration. Amide I and amide II bands are two major bands of the protein infrared spectrum. The amide I band (between 1600 and 1700 cm −1) is mainly associated with the C=O stretching vibration (70–85%) and is directly related to the backbone conformation. Amide II results from the N-H bending vibration (40–60%) and from the C–N stretching vibration (18–40%). This band is also conformationally sensitive^28^. Amide III is a very complex band resulting from a mixture of several coordinate displacements^29^. The further bands, amide IV to VII, are outside our measurement range. We measured along the fiber as presented in Fig. 2a.

Figure 2b presents a focal plane array (FPA) image of a selected area of the fiber obtained by the integration of the amide I signal of proteins. The infrared profile does not match perfectly the optical one (Fig. 2a) as the sample was not flat and some parts of the fiber were on different focal planes. Nevertheless, the extracted average spectrum presented in Fig. 2c is very similar to a protein. The amide I peak is centered at 1665 cm^−1^, marginally blue-shifted with respect to a reference value of ~1657 cm^−1^, but this can be due to misfolding of the proteins to a more compact state. By analyzing the spectra in 2^nd^ derivative (Suppl Fig. 1) two additional components at 1685 and 1638 cm^−1^ were detected, assignable to misfolded aggregates. By removing some spectral contribution arising from the surrounding material, the protein nature of the fiber is clearer and can be identified as a collagen-based fiber. Stani *et al*.^30^ confirmed that, upon aging, collagen loses its typical triple α-helix conformation in favor to a tighter aggregation, often associated with the increase of hydroxyproline cross bonding. Moreover, it has also been reported that aging affects the amide I to amide II ratio, as seen in Fig. 2c, lowering the signal of amide II and increasing of the signals in the 1100-1000 cm^−1^ spectral region, as a consequence of the formation of AGEs (Advanced Glycation Endproducts)^31^. Clearly, aging and signal artifact due to the sampling geometry affected the spectral quality and peak position. Nevertheless, analyses on the fiber strongly suggest that it is derived from collagen and, by definition, has an animal origin. Peaks comparable with those present in literature for similar samples in both amides and carbohydrate spectral region confirm this hypothesis.

**Figure 3:**
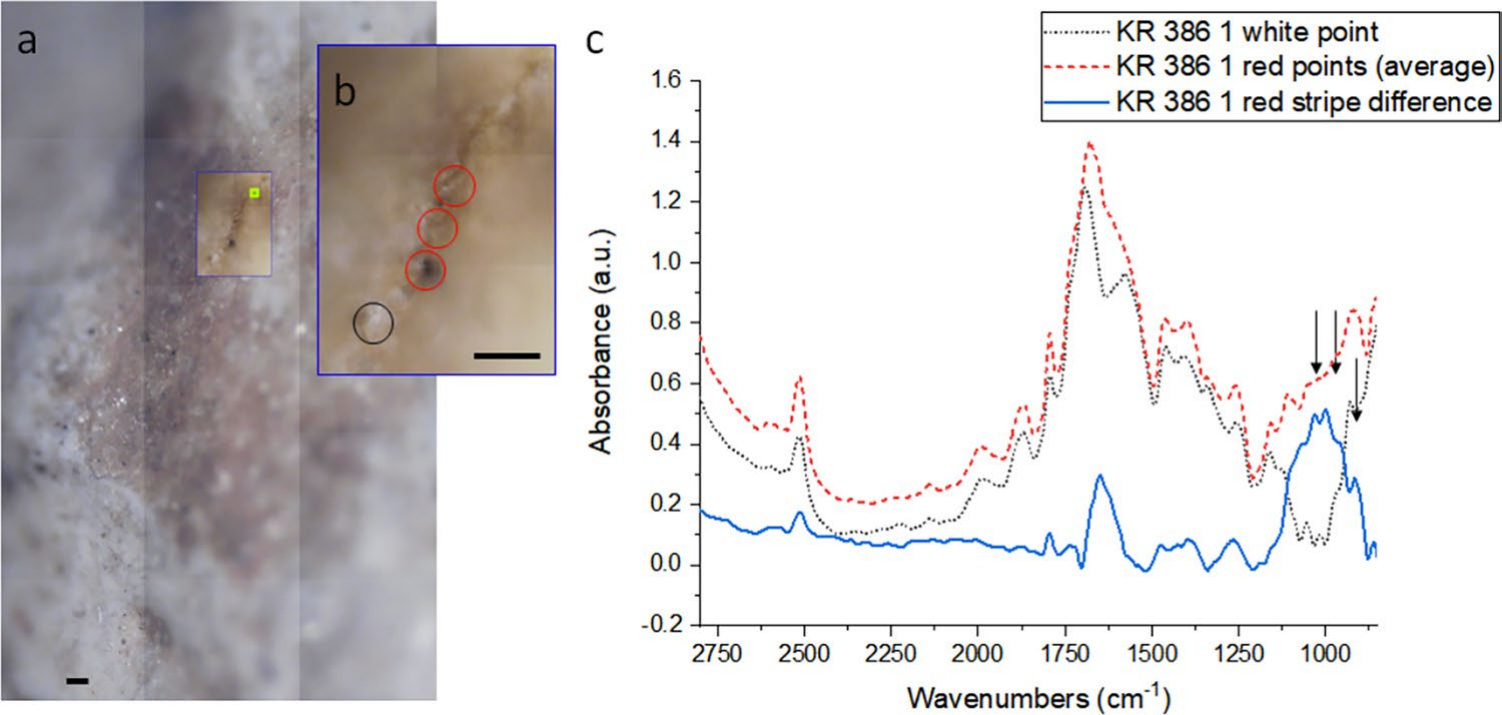
Optical image for red pigments. (**a**) Optical image obtained with a 4x objective of an area of interest of the talon 386.1. (**b**) Zoom of the measured area with a 15x Cassegrain objective. (**c**) spectra of the white spot in panel b (red circle - black dot line) and average spectrum of the three red spots in panel b (red circles - red dashed line), the blue line is the spectral difference between red and white areas. Arrows point at the three main bands of ochre. Scalebars 100 microns.

Several other areas were analyzed to understand the possible origin of stains and coloured marks present in the talon. In talon KR 386.1 a black patch was located 3.40 mm proximally from the tip of the talon, dorso-laterally. A reddish area was located at the proximal end of the talon, on the same side as the cut mark and the fiber, at the lateral edge of tuberculum extensorium. (Fig. 1) To highlight the differences of these spots with respect to the surrounding areas, the acquired data were divided by those in the proximity of the coloured areas. The acquired data are often noisy and the main bands for the chemical identification saturated, but in some cases it is possible to identify the compound using overtones or weak bands, that in the sample are strong absorbers and become intense. This is the case of the reddish stripe in Fig. 3a,b. The first point was collected in the white area and other three following the red line. From the spectra in Fig. 3c, it is possible to observe that all the data present the typical overtone peaks of carbonates at 2510 and 1790 cm^−1^. An abrupt signal loss above 2800 cm^−1^ (data truncated) can be due to a strong absorbance of the -OH moiety that saturates the band, suggesting the presence of a hydrated carbonate. The blue line in Fig. 3c is the spectral difference between the white area (black circle in the panel 3b) and the reddish areas (red circles in the panel 3b). From this plot it is possible to observe a strong, broad band from 1230 to 850 cm^−1^. By comparing this signal to spectral databases^32,33^, it can be identified as a mixture of red and yellow ochre, hence the orange color. This is a unique find at Krapina as Gorjanović-Kramberger^5^ reports no evidence of any kind of ochre at the site and we find no traces in the archaeological collections in the Croatian Natural History Museum. Given the sandstone nature of the site, ochres of any kind would not be expected to occur naturally. While there are more Krapina talons, at present, through IR analysis we have only analyzed talon 386.1. Future IR work may find additional evidence for the application of pigments on the surfaces of other Krapina talons.

**Figure 4:**
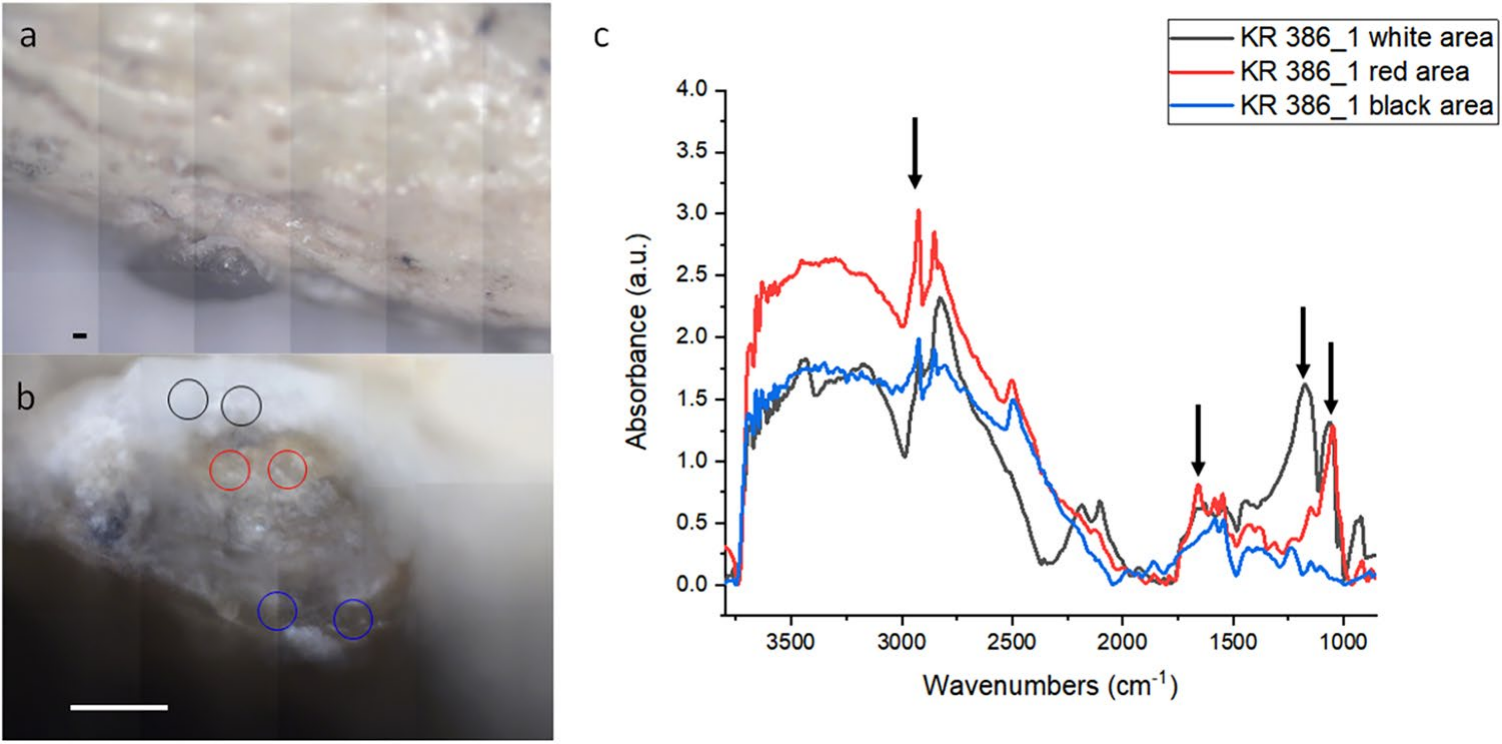
Optical image for black pigment. (**a**) Optical image obtained with a 4x objective of an area of interest of the talon 386.1. (**b**) Zoom of the measured area with a 15x Cassegrain objective. (**c**) Spectra of the spots in panel b, color code matching. Scalebars 100 microns. Arrows point to peaks of interest.

Another interesting feature is a black excrescence that can be found along the tip of the talon. From Fig. 4a,b, it can be seen that there are three areas in this part: one is the white area, another a red-brown intermediate and another a black zone. In this spot the scattering is quite intense and due to this phenomenon, the spectra are heavily affected by artifacts, like a strong non-linear baseline and peak inversion. To partially correct for these phenomena, the spectra were converted in reflectance and then a Kramers Kronig transform was performed to retrieve the absorbance. Figure 4c reviews the average spectra of the three areas. The white area is characterized by a strong double peak at 1175 and 1070 cm^−1^, probably due to the hydroxyapatite present in the bone material (spectrum IPR00041 Infrared and Raman Users Group (IRUG) database). Moving away from these sampling points, this signal decreases. The red-brown area instead has a spectrum similar to kaolin plus some charcoal contaminant (IMP00058 from IRUG). The black area presents no strong signals in the fingerprint region, but its spectrum is similar to coal tar (INR00205 IRUG database). So at least for this area no manganese oxide is present. The major part of the measured areas present peaks from the CH^-^ stretching at 2925 and 2850 cm^−1^, along with the signal at 1730 cm^−1^. These three signals are usually assigned to fatty acids indicating human handling. Given that we cannot be sure whether they are a contamination due to recent or ancient manipulation, these signals will not be further assessed this time.

## Discussion

Application of new non-invasive technological analyses is shown here to be an additional, effective tool in confirming Neandertal manipulations of a white-tailed eagle talon. Infrared spectrometry confirmed that the analyzed talon preserves traces of a collagen band, two different kinds of ochre and charcoal. A silicate coating, formed by natural diagenesis in the sandstone rock shelter environment, covers the most superficial layer of the talon, overlying the fiber and pigmented areas. This silicate layer seals the evidence under it and confirms the antiquity of different traces detected below. Within the wide cut mark at the proximal end of the analyzed talon, a fiber-like, twisted, dark-coloured strain was visible under low magnification. Infrared spectrometry revealed the fiber to be constituted of animal protein. Visible intra- and inter-strand aggregation within the analyzed area of the fiber indicate the fiber is collagen losing its original triple α-helix conformation, a consequence of diagenetic aging. This is additional confirmation of the antiquity of the animal fiber. An eagle origin of the collagen strand is unlikely since the talon appears to have been detached from the rest of the foot bones for some time, based on the spatial arrangement and orientation of the cut marks, other indications of anthropogenic modifications like nicks on the proximal edges and multiple wear traces along the talon. Thus, we consider the collagen strand is most likely a remnant of the leather or sinew string binding it together with the other talons. The fiber may be some other kind of animal tissue, possibly something else related to binding the talons together or another element, like a feather, added to the ornament for additional decoration. While it is not unexpected that the talons were bound together in prehistoric times, it is unexpected to find preserved remnants of the binding or other elements of the ornament.

Additionally, there are ochre traces found under the silicate coating. The yellow and red ochres would not be naturally occurring in the cave and we interpret the presence of these ochres a result of intentional modification by Neandertals. Ochre traces found on personal ornaments are usually interpreted as evidence of symbolic use of objects^34–38^. Close to the talon’s tip, traces of charcoal mixed with kaolin are found. The talon tip is broken with wear traces, which in combination with adjacent pigments could possibly further indicate use of pigments by the Krapina Neanderthals. Both black and red pigments are known to be used for symbolic purposes, possibly body paint and cave painting at various sites, including within exclusively Neandertal contexts^39–41^. The black pigment is commonly of manganese oxide origin, but this is not detected on this talon. Rather, charcoal traces on the talon imply that the talon came into contact with this black pigment. It is possible the charcoal pigment comes from deposits in the cave since Gorjanović-Kramberger reports a hearth in the top level at the site^1,42^. It is also possible the black was intentionally rubbed onto the talon. At present, it is not possible to determine if the presence of the black pigment was intentional or accidental. However, identification of red and yellow ochre on the talon clearly indicates human agency.

Analyses of these traces by IR spectrometry confirms that there is strong evidence of human agency modifying the talon. The anthropogenic collection of the Krapina eagle talons and their use as a type of jewelry were previously established^8^. Other evidence inspected here by the infrared spectrometry supports our earlier argument that this combination of traces should be interpreted as indicative of symbolic use of the talons by the Neandertals, especially the application of two types of ochre and the possible application of black pigment. A small remnant of an animal fiber further suggests the talon was bound into an assemblage. These non-invasive technologies offer important new ways to test the hypothesis of Neandertals using objects for symbolic purposes.

## Methods

All samples used in this study were covered by a silicate coating laid down in prehistory, presumably shortly after the talon was lost in the sediments. The nature of the sample and the fiber’s geometry imposed several constraints on the measurement approach that could be used to characterize it. At first we tried Attenuated total reflection (ATR) in microscopy, but the fiber was not flat enough to provide good contact with the germanium tip, and therefore it was not possible to obtain any data with this approach. Hence, it was decided to try transflection geometry, shining IR light onto the sample and collecting the reflected, or better, diffused light. This approach allowed obtaining some data, although the measured spectra were characterized by artifacts due to dispersion, scattering and with a quite low signal to noise ratio. All these issues in the sampling and signal quality directly reflected an intrinsic difficulty in the analysis and interpretation of the data.

Samples were measured at SISSI beamline at Elettra Sincrotrone Trieste, Italy^43^. Due to sample size and morphology, the selected sampling technique was transflection. Distinctive areas of talon 386.1 were measured in spectroscopy and mapped either using conventional source or using Synchrotron Radiation (SR). Spectroscopic data were obtained by closing the knife-edge apertures of the Hyperion 3000 microscope (Bruker Optik GmbH) at 50×50 µm and by averaging 512 scans, at 40 kHz scanner speed, with a spectral resolution of 4 cm^−1^. A single point detector MCT (mercury-cadmium-telluride) was used. SR mapping was obtained by closing the apertures at 20×20 µm and collecting a spectrum every 10 microns accumulating 1024 scans at 120 kHz scanner speed. FTIR (Fourier Transform Infrared Spectroscopy) imaging was carried out using a detector array (Focal Plane Array, FPA) with 64×64 sensitive elements, over an area ~150×150 µm; the image was obtained after averaging 1024 scans at 5 kHz scanner speed. All acquired spectra were corrected for water vapor and CO_2_ contribution using the OPUS routine. Several chemical maps were obtained by the integration of signals of proteins.

### Ethics declarations

This work was entirely supported by internal funds.

## Supplementary information

Supplementary information

## Data Availability

Talon 386.1 and the other seven talons and phalanx are available for study in the Croatian Natural History Museum, Zagreb.
