# Supplementary material for: Surface analysis of an eagle talon from Krapina
Source: Sci Rep. 2020 Apr 14;10:6329. doi: 10.1038/s41598-020-62938-4 (PMC7156502; doi:10.1038/s41598-020-62938-4)
Supplement: Supplementary file 1 — Supplementary information [file 41598_2020_62938_MOESM1_ESM.pdf]

Surface analysis of an eagle talon from Krapina

Davorka Radovčić, Giovanni Birarda, Ankica Oros Sršen, Lisa Vaccari, Jakov Radovčić & David W. Frayer

Supplementary Figure 1 Optical image for fiber. a) In red the area raster scanned with the infrared synchrotron (IRSR)beam, a 10x8 pixel map was measured closing the apertures at 20x20 micron, sampling every 10 microns. b) a false color map obtained by integrating the amide I and amide II bands of the IRSR data. c) in black the average absorbance spectrum of the pixels on the diagonal of the measured map, in red its second derivative, with arrows pointing to the main components of the amide I, discussed in the main text.

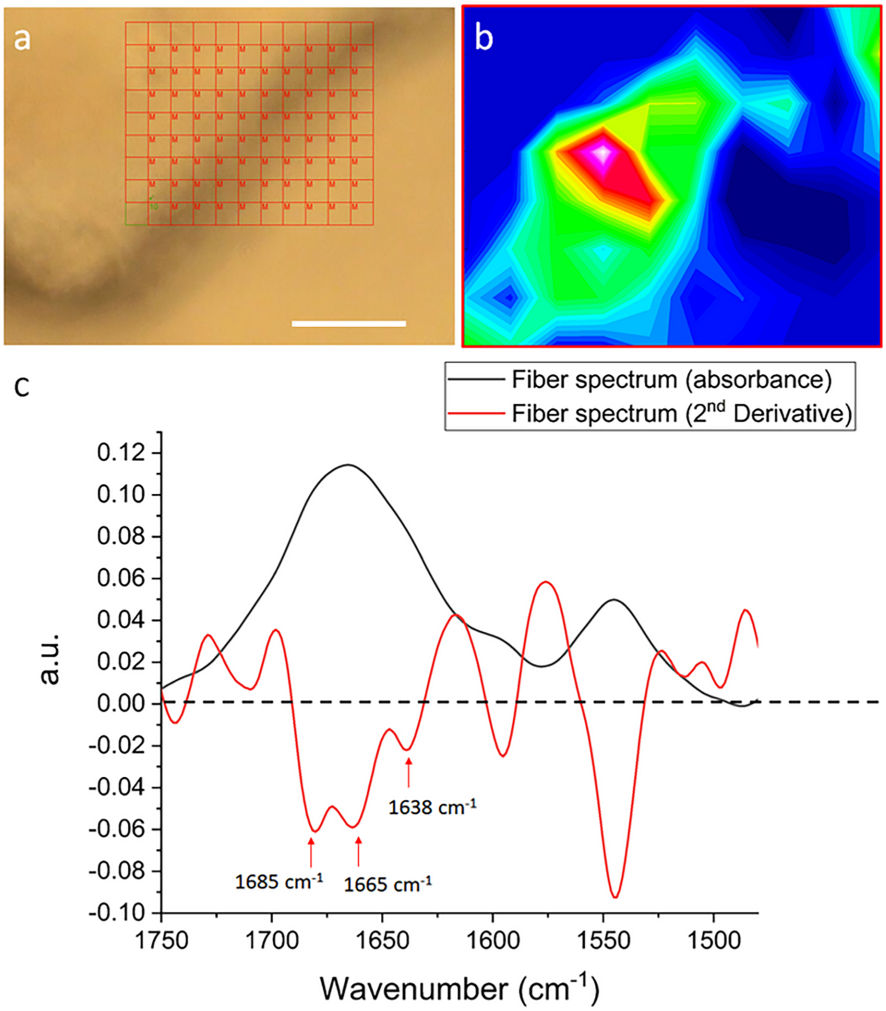

a
b
c
Fiber spectrum (absorbance)
Fiber spectrum (2nd Derivative)
0.12
0.10
0.08
0.06
0.04
0.02
0.00
-0.02
-0.04
-0.06
-0.08
-0.10
a.u.
1685 cm-1
1665 cm-1
1638 cm-1
1750
1700
1650
1600
1550
1500
Wavenumber (cm-1)
